# Pharmacokinetics of flunixin meglumine in mature swine after intravenous, intramuscular and oral administration

**DOI:** 10.1186/1746-6148-9-165

**Published:** 2013-08-13

**Authors:** Monique D Pairis-Garcia, Locke A Karriker, Anna K Johnson, Butch Kukanich, Larry Wulf, Suzanne Sander, Suzanne T Millman, Kenneth J Stalder, Johann F Coetzee

**Affiliations:** 1 Department of Animal Science, Iowa State University, Ames, IA 50011, USA; 2 Swine Medicine Education Center, College of Veterinary Medicine, Iowa State University, Ames, IA 50011, USA; 3 Department of Anatomy and Physiology, College of Veterinary Medicine, Kansas State University, Manhattan, KS 66506, USA; 4 Pharmacology Analytical Support Service, College of Veterinary Medicine, Iowa State University, Ames, IA 50011, USA; 5 Veterinary Diagnostic and Production Animal Medicine and Biomedical Science, Iowa State University, Ames, IA 50011, USA

**Keywords:** Swine, Gilt, Lameness, Flunixin meglumine, Pharmacokinetics, NSAIDs, Oral bioavailability

## Abstract

**Background:**

The purpose of this study was to determine intravenous (IV), intramuscular (IM) and oral (PO) FM PK in mature swine. Appropriate pain management for lameness in swine is a critical control point for veterinarians and producers, but science-based guidance on optimal housing, management and treatment of lameness is deficient. Six mature swine (121–168 kg) were administered an IV, IM, or PO dose of flunixin meglumine at a target dose of 2.2 mg/kg in a cross-over design with a 10 day washout period between treatments. Plasma samples collected up to 48 hours post-administration were analyzed by high pressure liquid chromatography and mass spectrometry (HPLC-MS) followed by non-compartmental pharmacokinetic analysis.

**Results:**

No adverse effects were observed with flunixin meglumine administration for all routes. Flunixin meglumine was administered at an actual mean dose of 2.21 mg/kg (range: 2.05-2.48 mg/kg) IV, IM and PO. A mean peak plasma concentration (C_MAX_) for IM and PO administration was 3748 ng/ml (range: 2749–6004 ng/ml) and 946 ng/ml (range: 554–1593 ng/ml), respectively. T_MAX_ was recorded at 1.00 hour (range: 0.50-2.00 hours) and 0.61 hours (range: 0.17-2.00 hours) after PO and IM administration. Half-life (T ½ λ_z_) for IV, IM and PO administration was 6.29 hours (range: 4.84-8.34 hours), 7.49 hours (range: 5.55-12.98 hours) and 7.08 hours (range: 5.29-9.15 hours) respectively. In comparison, bioavailability (F) for PO administration was 22% (range: 11-44%) compared to IM F at 76% (range: 54-92%).

**Conclusions:**

The results of the present study suggest that FM oral administration is not the most effective administration route for mature swine when compared to IV and IM. Lower F and Cmax of PO-FM in comparison to IM-FM suggest that PO-FM is less likely to be an effective therapeutic administration route.

## Background

Researchers have reported that lameness is a major factor when culling females from the swine breeding herd [1-3]. Lameness in breeding aged swine has a large negative economic impact to livestock producers [4] and has been highlighted as a welfare concern [5]. Lameness was ranked as the third most common reason for culling sows, comprising 15% of the cull market in the U.S. [6]. In addition leg soundness was identified as the most common involuntary reason for culling sows [7]. Lameness can be caused by neurological deficits, lesions of the hoof and/or limb, mechanical-structural conformation, trauma, or metabolic and infectious disease [8,9]. Appropriate management of pain, regardless of etiology is a critical control point for veterinarians and producers [10]. However, science-based guidance for the industry on optimal housing, management and treatment of lame swine is deficient. There are currently no approved analgesia drug treatments for lame swine in the U.S. [11]. Research to address the limited knowledge in this area is essential to formulating science-based recommendations for swine producers. In addition, providing appropriate drug regimens will allow caretakers and veterinarians to manage pain effectively on farm [10,12].

Flunixin meglumine acts by decreasing prostaglandin synthesis by inhibiting the enzyme cyclo-oxygenase (COX; a major player in the inflammatory process). Flunixin meglumine has been shown to be effective in managing pain associated with a variety of companion animal diseases [13,14]. Pharmaceutical advantages for FM include its potency as a COX inhibitor and effective analgesic strength for acute pain [15].

Flunixin-meglumine (FM) is approved in the US as an intramuscular (IM) injection in swine, and is available for intravenous (IV) and oral (PO) administration in other species [11]. Flunixin meglumine has been used exclusively within the veterinary community and is labeled for use in the U.S. for beef cattle, dairy cattle, horses and swine [16]. Flunixin Meglumine is labeled for pyrexia control associated with swine respiratory disease at 2.2 mg/kg dose administered IM [17]. However, this drug’s analgesic effects at the recommended dose have not been quantified. The half maximal effective concentration (EC_50_) of a drug is often used to measure a drug’s potency. Although there are currently no studies that have been conducted on EC_50_ of FM in swine, a study identifying FM EC_50_ between 0.2-0.9 ug/ml in an arthritis model in horses [18] may provide a guideline to determine FM concentrations required to provide analgesia in swine.

Although FM is not specifically labeled for pain management in swine, it can be used to alleviate pain in pigs under the U.S. Animal Medicinal Drug Use Clarification Act (AMDUCA).

Pharmacokinetic (PK) parameters for FM have been evaluated in several species including cattle [19], small ruminants [20,21], horses [18], chickens [22], and companion animals [23]. To the authors’ knowledge there have been two peer-reviewed articles published on FM PK in swine [16,24]. However, both studies evaluated FM-PK properties in prepubertal swine weighing between 18.6 and 40 kg, and neither study evaluated oral bioavailability (F) of FM. The purpose of this study was to determine IV, IM and oral FM PK in mature swine.

## Results

No adverse effects on the sow were observed following IV, IM or PO-FM administration and drug levels were below the limit of detection on baseline days suggesting that the 10-day washout period provided adequate time between rounds to prevent residual drug effects. Weight and round had no effect (P > 0.05) but time-point, route and time-point*route interaction were different (P < 0.002).

Purity analysis conducted in the lab on the oral powder resulted in a flunixin concentration of 34 mg Flunixin/g sample ± 10 mg Flunixin/g sample (or 3.4% ± 1% of the powder). We analyzed the data using flunixin and not flunixin meglumine, thus our results may be within 3-5% higher or lower than stated results. Utilizing the concentration presented on the label, sows received an actual mean dose of 2.21 mg/kg (range: 2.05-2.48 mg/kg). Utilizing the results from our purity analysis sows received an actual mean dose of 3.02 mg/kg (range: 2.04-4.36 mg/kg).

Bioavailability and mean peak plasma concentration (Cmax) was greater for IM-FM compared to PO-FM (P < 0.002), but time to Cmax (Tmax) was not different (P > 0.05) between routes. Mean residence time extrapolated to infinity (MRT 0-INF) was greater in IV-FM compared to PO and IM but PO-FM and IM-FM were not different (P > 0.05). Clearance per fraction of the dose absorbed (Cl-F) was greater in PO-FM compared to IV and IM-FM (P = 0.0004). Route had no effect on elimination half-life (T ½ λz; P > 0.05).

Table 1 presents the mean plasma concentrations by time for IV, IM and PO-FM administered at 2.2 mg/kg.

**Table 1:** **Mean** (± **SD**, **n** = **6**) **flunixin meglumine plasma concentrations** (**ng ml**^-**1**^) **after a single intravenous** (**IV**), **intramuscular** (**IM**) **or oral** (**PO**) **administration in gilts**

**Administration route**						
**Time (hours)**	**IV**	**±SD**	**IM**	**±SD**	**PO**	**±SD**
**0**.**05**	21695.1	4036.6	-	-	-	-
**0**.**10**	18615.7	3638.1	-	-	-	-
**0**.**17**	22819.35	17414.12	2830.6	1237.3	-	-
**0**.**25**	-	-	-	-	537.4	231.4
**0**.**33**	10018.9	1896.4	3100.0	1224.0	-	-
**0**.**5**	8989.7	3304.1	-	-	987.8	432.9
**0**.**67**	-	-	3258.6	1565.2	-	-
**1**.**0**	3358.1	829.9	2761.1	850.1	786.2	385.1
**2**.**0**	2005.4	663.1	2264.1	380.4	869.7	337.2
**4**.**0**	908.9	254.5	1194.8	574.3	440.2	204.1
**8**.**0**	407.1	87.6	593.2	327.0	173.7	114.7
**12**.**0**	-	-	-	-	76.2	61.4
**16**.**0**	93.8	57.4	176.1	87.2	46.7	38.8
**24**.**0**	32.8	20.4	66.2	30.2	16.7	16.5
**36**.**0**	10.8	7.28	28.4	17.2	6.15	4.08
**48**.**0**	2.68	1.80	10.2	8.8	2.2	1.1

Time (hours) began once drug was administered.

Table 2 summarizes the calculated PK for IV-FM, IM-FM and PO-FM. The geometric mean T ½ λz was 6.29 hours (range 4.84 to 8.34 hours) for IV-FM resulting from a geometric mean Vz of 0.914 L/kg, (range 0.614 – 1.38 L/kg) and a geometric mean Cl of 1.68 ml/min/kg, (range 1.40 – 2.54 ml/minute/kg). . The geometric mean C_max_, T_max_, and T ½ λz for PO-FM were 946 ng/mL (range 554–1593 ng/mL), 1.00 hour (range 0.50 – 2.00 hours), and 7.1 hours (range 5.29–9.15 hours) respectively. Mean F and mean absorption time (MAT) were determined to be 22% (range 11– 44%) and 2.57 hours (range 2.18–2.67 hours). The geometric mean C_max_, T_max_, and T ½ λz for IM-FM were 3748 ng/mL (range 2749–6004 ng/mL), 0.61 hours (range 0.17-2.00 hours), and 7.49 hours (range 5.55–12.98 hours). Mean F and MAT were determined to be 76% (range 54-92%) and 3.4 hours (range 2.23–6.03 hours).

**Table 2:** **Geometric mean** (± **SD**, **n** = **6**) **flunixin meglumine pharmacokinetic parameters after a single intravenous** (**IV**), **intramuscular** (**IM**) **or oral** (**PO**) **administration in gilts** (**2**.**2 mg**/**kg **^-**1**^)

**Parameter**	**Units**	**Administration Route**					
		**IV**	**±SD**	**IM**	**±SD**	**PO**	**±SD**
**AUC **_**EXTRAP**_	%	0.1	0.08	0.5	1.2	0.6	0.29
**AUC **_**INF**_	h*ng/ml	21635	3966.7	16849	4257.7	4836	2333.0
**C0**^**d**^	ng/ml	24960	5378.2	-	-	-	-
**Cl**^**e**^	h	1.68	0.38	-	-	-	-
**T** ½ **λz**	h	6.29	1.03	7.49	2.54	7.08	1.33
**λz**	1/h	0.11	0.01	0.09	0.03	0.10	0.02
**MRT 0**-**INF**	h	3.01	0.63	6.41	1.85	5.58	0.86
**Vss**	l/kg	0.30	0.07	-	-	-	-
**Vz**	l/kg	0.91	0.26	-	-	-	-
**CMax**	ng/ml			3748	1110.8	946	401.2
**TMax**	h			0.61	0.59	1.00	0.75
**F**		-	-	0.76	0.02	0.22	0.11

Flunixin meglumine noncompartmental pharmacokinetics (WinNonlin 5.2, Pharsight Inc. Cary NC, USA). *AUC extrapolated* percent of the AUC extrapolated, *AUC*_*INF*_ area under the curve extrapolated to infinity, *CMAX* maximum plasma concentration, *T ½ λz* terminal half-life, *λz* terminal rate constant, *MRT* mean residence time extrapolated to infinity, *TMAX* time to C_MAX_, *F* fraction of the dose absorbed.

## Discussion

In this study, we evaluated the PK parameters of IV, IM and PO-FM in 42-week old mature swine (152–168 ±11.3 kg) including F. The administrative dosages for all routes were selected as the labeled IM dose for swine at 2.2 mg/kg. Although FM PK properties were previously evaluated at 2.2 mg/kg in swine [24], the authors completed this work using younger, immature pigs (8-weeks of age) and did not evaluate PO-FM administration. Hence, the current study is novel for evaluating mature pigs and a different administration route using 2.2 mg/kg dose.

Intravenous FM administration is imperative in PK studies as a means to determine F for non-IV routes. Elimination half-life was not different between administration routes and T ½ λz results were similar compared to both PK studies evaluating IV-FM in swine [16,24]. The MRT at 3.01 hours and Cl at 1.68 ml/min/kg were lower than results reported by Buur and colleagues [16] and Yu and colleagues [24]. Although the cause of these differences is unknown, variation between older and younger swine may be attributed to difference in genetic lines, body condition, or immune function. Further research addressing how these factors impact drug distribution and elimination should be conducted.

However, intravenous administration is difficult to perform on swine due to inaccessible superficial veins and thick subcutaneous fat layers [25]. For all ages and weights, the two most common methods for IV administration on farm include 1. Temporary aural vein catheterization [26] or 2. IV jugular vein injection using a “blind stick” approach, during which blood is collected without visualization of the vein [27,28]. Aural vein catheterization requires a veterinarian and is an unrealistic option for daily on-farm treatment. Blind-stick injection can be performed by farm personnel, but is unreliable as it is difficult to ensure that drug is not administered extravascularly. Thus, IV administration of FM is impractical for on-farm use.

Possible explanations for such variation in Cmax and lower oral F include differences in drug formulations, delivery methods and species variation [22]. Compounded drugs in veterinary medicine do not undergo the same regulatory quality control to assure drug purity, potency and stability as compared to FDA approved drugs [11]. Due to the nature of compounded drugs, a purity analysis was performed. The company distributing the flunixin powder stated 25 mg of flunixin meglumine/ gram of powder. The results of our purity analysis concluded 35 mg ± 10 mg of flunixin/ gram of powder. Differences between results may be attributed to use of the flunixin compound as a standard compared to flunixin meglumine or variation with equipment. However, in vivo dissolution of the drug from the formulation provided by the company may also vary resulting in a low oral F.

The form of the drug may have also greatly influenced oral F. Flunixin meglumine was administered orally as a fine powder mixed with cookie dough. It was difficult to determine if powder may have been lost or not consumed during administration as the powder was extremely fine and easily dispersed. This may have falsely lowered oral F if some pigs did not consume the entire dose of the drug. Previous studies evaluating PO-FM in goats, laying hens, broilers, and horses all utilized oral gavage as the administration method for FM [20,22,29,30]. This method, although effective, is difficult to perform on swine and does not represent a practical option for drug administration on farm. Pellegrinin-Masini and colleagues [31] demonstrated decreases in F when FM was mixed with molasses and administered on the back of the tongue with a syringe (purpose of method was to mimic clinical practice). Bioavailability with syringe administration in comparison to oral gavage dropped approximately 14% [30]. Other administration options that could have been utilized include mixing the FM powder with feed. However it should be noted that under current AMDUCA policies extra-label drug use in feed is illegal [11]. Because this is the first published report of PO-FM F in swine, we cannot rule out that the low oral F is due to species variation. Further studies utilizing different FM oral forms and administrative techniques (oral gavage, drug-feed mixture) in swine should be performed.

By definition, the “half maximal effective concentration (EC_50_) refers to the concentration of a drug, antibody or toxicant which induces a response halfway between the baseline and maximum after some specified exposure time. It is commonly used as a measure of drug's potency” [32]. No studies have been conducted on FM EC_50_ in swine but a study conducted in the horse determined the EC_50_ of FM to be between 0.2-0.9 ug/ml [21] determined FM EC_50_ in horses using a chemically induced arthritis model and evaluating thromboxane A generation (an index for NSAID therapeutic effect in horses; [33]. Although inter-species extrapolations of EC_50_ drug values may not always be accurate, data generated from this chemically induced arthritis model in the horse may be used as an initial starting point to predict the drug concentration required to provide effective analgesia in lame swine. In this present study, a single dose of flunixin at 2.2 mg/kg administered either IM or IV resulted in sustained drug concentrations > 0.2 ug/ml for up to 8, whereas FM administered PO sustained these effective drug concentrations for only 4 hours. It should be noted that IM-FM administration yielded an average drug concentration of 0.18 ug/ml at 16 hours post administration suggesting that an Ec50 > 0.2 ug/ml may be achieved between 8 and 16 hours.

## Methods

This study was approved by the Institutional Animal Care and Use Committee at Iowa State University.

### Animals and housing

Six healthy 42-week old Newsham genetic gilts (152–168 ±11.3 kg) were used for this study. Gilts were confirmed to be healthy by physical examination by a veterinarian. Gilts were obtained from a commercial production unit in Iowa and were acclimated to personnel for 7 days prior to study commencement. When off trial (defined as gilts not receiving the drug and having no blood collected), gilts were housed in individual pens. Each pen measured 3.7 m length × 1.4 m width × 1.2 m height and had a solid concrete floor with a rubber mat (2.4 m length × 2 cm height × 1.4 m width). Metal fences (1.2 m height × 76 cm width) were affixed to the end of each home pen. Gilts were provided *ad libitum* access to water via one nipple drinker (Trojan Specialty Products Model 65, Dodge City, KS). Gilts were hand-fed a custom mixed diet free of antibiotics or medications composed of corn, soybean meal and soy hulls, designed to meet or exceed nutrient requirements for gilts. Approximately 1.8 kg of feed was fed at 0800 and 0.45 kg of feed was fed at 1600 onto a raised concrete step (55 cm length × 55 cm in width × 24 cm). Matrix (Altrenogest formulation; Intervet/Schering-Plough, Milsboro, DE- Dose: 6.8 ml-15 mg) was added to one kg of feed daily to prevent estrus cycle initiation.

Twenty-four hours before study commencement, gilts were moved to individual gestation stalls (2.1 m length × 0.6 m width) with nonslip rubber flooring. Gilts had access to the same type of nipple drinker previously described for the pen, and gilts remained in their stalls for a total of 72 hours while on trial (on trial defined as gilts receiving drug and having blood collected). Gilts on trial regardless of administration route were fed on the same schedule with the following ration: **Day 1**: 0.22 kg at 6:30, 0.45 kg at 8:50, 1.36 kg at 9:20, and .45 kg 16:15; **Day 2**: 1.8 kg at 8:15 and 0.45 kg at 16:15. All gilts returned to their individual pens between trials. Lights were on a 12:12 light dark cycle (light hours [0600 and 1800]).

Attitude, appetite, and blood collection sites of sows were monitored twice daily during each study period and each sow was assessed for adverse reactions to drug administration. Post-mortem injection site tissue damage was not evaluated.

### Study design

A cross-over design study [34] was conducted over three rounds such that all sows received each administrative route. Gilts were blocked by body weight and randomly allocated to one of three administration routes for the first round. Using the closest available data on FM in swine, a 10-day washout period was chosen as it was greater than 30 times the half-life reported in swine [16,24]. Gilts were weighed 20 h before initiating the study and these weights were used to calculate drug dosages. For oral administration, the dose was rounded to the nearest whole gram. For intravenous and intramuscular administration, the dose was rounded to the nearest half or whole milliliter.

In the first round, two gilts were administered an intravenous injection of FM (**IV**-**FM**) at 2.2 mg/kg (Banamine-S® 50 mg/ml solution for injection; Schering-Plough Animal Health Corp., Union, NJ, USA Lot # 1155103) as a single bolus injection in the right jugular vein using a 25.4 mm 16 gauge hypodermic needle. Two gilts received FM per os (**PO**-**FM**) at 2.2 mg/kg (Flunixin meglumine 500 mg/20 gm apple flavored powder packet for oral administration; Wedgewood Compounding Pharmacy, Swedesboro, NJ, USA Lot # 2011908@2). Powder was mixed with approximately 24 g of sugar cookie dough (sows had been previously trained using cookie dough as a positive reinforcement), divided into three, 8 gram round balls, and administered in a clean feeding bowl. An intramuscular injection (**IM**-**FM**) of 2.2 mg/kg flunixin meglumine was given to the remaining two gilts (Banamine-S® 50 mg/ml solution for injection; Schering-Plough Animal Health Corp., Union, NJ, USA Lot # 1155103). Drug was administered as two individual injections no greater than 5 ml volume into the neck muscles using a 77 mm 18 gauge needle. This process was repeated over two additional rounds so that all gilts received all FM routes. The experimental unit was the individual gilt (n = 6/treatment). This experimental design provided robust control of intra- and inter-animal physiological response variations reduced the experimental units (gilts) required.

### Blood collection

All blood samples (9.0 mL/sample) were collected via the left or right jugular vein using a 25.4 mm 16 gauge hypodermic needle (Air-Tite Products, Virginia Beach, VA, USA) and 12 ml luer lock syringe (TycoHealth Care, Mansfield, MA, USA). During blood collection, gilts were manually restrained using a pig snare. Blood was collected from gilts receiving IV-FM at 0.05, 0.1, 0.17, 0.33, 0.5, 1, 2, 4, 8, 16, 24, 36 and 48 hours after drug administration. For time-point 0.05 hours and 0.1 hour, blood was collected from the left jugular vein that was not used to administer the drug. Blood was collected from gilts receiving PO-FM at 0.25, 0.5, 1, 2, 4, 8, 12, 16, 24, 36, and 48 hours after PO administration. Blood was collected from gilts receiving IM-FM at 0.17, 0.33, 0.67, 1, 2, 4, 8, 16, 24, 36, and 48 hours after IM injection. A baseline sample was collected 20 hours prior to drug administration for all routes. Samples were immediately transferred to a sodium heparin 10 ml blood collection tube (BD Vacutainer, Franklin Lakes, NJ, USA) and stored on ice before processing. Blood samples remained on ice for no longer than 130 minutes prior to centrifugation for 10 minutes at 1,500 *g*. Collected plasma was placed in cryovials and frozen at −70°C until analysis. All samples were analyzed within 70 days after sample collection and within 3 consecutive days once analysis began.

### HPLC/MS analysis of FM concentrations

Plasma FM concentrations were determined using high-pressure liquid chromatography (Surveyor MS Pump and Autosampler, Thermo Scientific, San Jose, CA, USA) and mass spectrometry (TSQ Quantum Discovery MAX, Thermo Scientific, San Jose, CA, USA). Plasma samples (0.20 ml) and the internal standard (Flunixin-d3; 40 ug (10 μL of 4.0 ng/ml)) were treated with 20 μL of 30% perchloric acid. Samples were vortexed for 5 seconds and centrifuged for 20 minutes at 2,500 × *g* to precipitate the sediment. The supernatant (~80 μL) was pipetted into a glass insert containing 120 μL of 1.9% ammonium hydroxide in 25% aqueous acetonitrile and fitted to an injection vial. The injection volume equaled 12.5 μL. Two mobile phases utilized were as follows: A. 0.1% formic acid in water B. 0.1% formic acid in an acetonitrile at a flow rate of 0.250 mL/min. The mobile phase began at 15% B with a linear gradient to 95% B at 7 minutes, which was maintained for 1.5 minutes, followed by a re-equilibration to 15% B. Separation was achieved with a solid-core c18 column (KinetexXB -C18, 100 mm × 2.1 mm, 2.6 μm particles, Phenomenex, Torrance, CA, USA) maintained at 40°C. Flunixin meglumine eluted at 6.94 minutes. Three SRM transitions were monitored for FM and 3 SRM transitions were used with the internal standard, FM-d3. The quantifying ions for FM were 109, 267, and 279 m/z. Sequences consisting of plasma blanks, calibration spikes, and FM plasma samples were batch processed with a processing method developed in the Xcalibur software (Thermo Scientific, San Jose, CA, USA). The processing method automatically identified and integrated each peak in each sample and calculated the calibration curve based on a weighted (1/X) linear fit. Plasma FM concentrations in unknown samples were calculated by the Xcalibur software based on the calibration curve. Results were then viewed in the Quan Browser portion of the Xcalibur software. The standard curve in gilt plasma was linear from 0.005 to 10 μg/mL. The coefficient of determination (R squared) exceeded 0.998 and all measured values were within 15% of the actual values with most of the values less than 7% difference from the actual values. The limit of quantification for this assay was determined to be 0.005 ug/mL, while the limit of detection was at 0.001 ug/mL.

### Pharmacokinetic analysis

Pharmacokinetic analyses for plasma flunixin concentrations over time were performed with computer software (WinNonlin 5.2, Pharsight Corporation, Mountain View, CA, USA) and analyzed using non-compartmental methods [35].The parameters included the area under the curve from time 0 to infinity (AUC_INF_) using the linear trapezoidal rule, percent of the AUC extrapolated to infinity (AUC _EXTRAP_), plasma clearance (Cl), first-order rate constant (λ_z_), terminal half-life (T_½_ λ_z_), apparent volume of distribution at steady state (Vss), apparent volume of distribution of the area (Vz), mean residence time extrapolated to infinity (MRT), and mean absorption time (MAT). The maximum plasma concentration (C_MAX_) and the time to maximum plasma concentration (T_MAX_) were observed for PO and IM administration. The concentration at time 0 (C0) was calculated by log-linear regression using the first two time points after IV administration. The AUC_EXTRAP_ was determined by multiplying the last measured plasma concentration by the λz. The range of the λz was determined by visual inspection of the plasma profile and determined by linear regression of time and natural log (ln) of the plasma concentration. The Vz was determined by dividing the drug dose by λz *AUC_INF_. The Vss was determined by multiplying the MRT by the Cl. The Cl was determined by dividing the dose by AUC. The F was estimated by dividing the oral (AUC/Dose) by the IV (AUC/Dose) for each individual animal. The MAT was estimated by subtracting the IV MRT from the PO MRT. A 13.9% variation within samples was detected as compared to the internal standard response across all samples.

### Purity analysis

Flunixin concentration in the powder (Wedgewood Compounding Pharmacy) was quantified using high performance liquid chromatography with mass spectrometry detection (Finnigan LTQ, Thermo Scientific, San Jose, CA, USA). The flunixin meglumine standard (Sigma Aldrich) and flunixin powder were dissolved in methanol and vortexed, heated, and sonicated to ensure complete dissolution of flunixin and then further diluted with 25% acetonitrile. Mobile phase consisted of a gradient of water and acetonitrile with a flow rate ramp starting at 0.265 mL/min and increasing to 0.3 ml/min. A C18 column (ACE 3 C18, 150 × 2.1 mm, 3 μm) was used for separation with elution occurring at 12.53 minutes. Mass spectrometry detection was achieved using electrospray ionization in positive ion mode as previously described.

### Statistical analysis

Data were analyzed using SAS software version 9.3 (SAS Institute Inc. Cary, NC, USA).

Drug concentrations were analyzed using PROC GLIMMIX procedures of SAS. The statistical model included the fixed effect of route (IV vs. IM v. PO), time point, round (1 vs. 2 vs. 3), timepoint*route interaction, and weight as a linear covariate. Sow was included as a random effect, and a repeated statement with sow as the subject was also used. Fixed effect least square means were separated using the PDIFF option in SAS. A *P*-value of ≤ 0.05 was considered to be significant for the GLIMMIX analysis of variance and when separating means.

## Conclusions

The results of the present study suggest that depending on specific formulation used, FM oral administration in swine may not provide the most effective administration route for mature swine when compared to IV and IM administration routes. Lower F and Cmax of PO-FM in comparison to IM-FM suggest that PO-FM clinical efficacy is predicted to be a less effective therapeutic administration route. If the EC_50_ for FM is similar in swine as was presented in the arthritis model in horses, FM may provide analgesic effects up to 8 hours after drug administration.

## Abbreviations

AUC extrap: Percent of the AUC extrapolated; AUCINF: Area under the curve extrapolated to infinity; Cl: Plasma clearance; C0: Concentration extrapolated to time 0 using log-linear regression of the first two time points; CMAX: Maximum plasma concentration; TMAX: Time to C_MAX_; T ½ λz: Terminal half-life; λz: Terminal rate constant; MRT: Mean residence time extrapolated to infinity; Vss: Volume of distribution at steady state; Vz: Volume of distribution, area method; MAT: Mean absorption time; F: Fraction of the dose absorbed; COX: Cyclo-oxygenase; HPLC-MS: High pressure liquid chromatography and mass spectrometry detection; PK: Pharmacokinetic; FM: Flunixin meglumine; NSAIDS: Non-steroidal anti-inflammatory drugs; IV: Intravenous; IM: Intramuscular; PO: Per os (by mouth).

## Competing interests

The authors declare that they have no competing interests. Dr. Coetzee has been a consultant for Intervet-Schering Plough Animal Health, Boehringer-Ingelheim Vetmedica and Norbrook Laboratories Ltd. Dr. KuKanich has been a consultant for Bayer Animal Health, Central Life Sciences, Pfizer Animal Health, and Procyon Pharmaceuticals. Dr. Millman has been a consultant for or has received funding from Boehringer-Ingelheim Vetmedica, Bayer Animal Health and Pfizer Animal Health. Dr. Johnson and Dr. Stalder have been a consultant for or have received funding from Boehringer-Ingelheim Vetmedica, Pfizer Animal Health and Elanco Animal Health. Dr. Karriker has been a consultant for or has received funding from Boehringer Ingelheim Vetmedica and Bayer Animal Health.

## Authors’ contributions

JFC and LAK conceived the study, participated in the design and coordination, performed the pharmacokinetic analysis and aided in drafting the manuscript. MPG participated in data compilation, sample processing, design and coordination and drafted the manuscript. AKJ provided funding participated in the design and coordination, and aided in interpretation of the data and drafting the manuscript. KJS, and STM participated in the design, coordination, interpretation of the data and drafting the manuscript. BK assisted with the pharmacokinetic analysis. LWW performed the plasma drug analysis on all samples. All authors read and approved the final manuscript.
